# Editorial: Beyond energy production: exploring mitochondrial dynamics and disease

**DOI:** 10.3389/fcell.2026.1872916

**Published:** 2026-06-02

**Authors:** Nahid A. Khan, Ruben Torregrosa-Muñumer

**Affiliations:** Stem Cells and Metabolism Research Program, Faculty of Medicine, University of Helsinki, Helsinki, Finland

**Keywords:** mitochondria, mitochondrial dynamics, mitochondrial disease, redox metabolism, metabolic signaling, mitochondrial quality control, cell fate regulation, mitochondrial therapy

## Introduction

Mitochondria are no longer viewed as static ATP-producing organelles, but rather as dynamic, stress-responsive signaling hubs integrating metabolism, quality control, inflammation, redox balance, cell fate decisions, and tissue adaptation. The articles collected in *Beyond Energy Production* reflect this conceptual shift, highlighting how mitochondrial biology extends far beyond ATP generation into disease mechanisms, adaptive remodeling, and therapeutic opportunity. Studies across cardiovascular biology, skeletal muscle aging, neurodegeneration, lung disease, and ischemia-reperfusion injury, position mitochondria as an active signaling center and not passive responders to damage. Together, these contributions highlight key emerging questions: how mitochondrial dynamics are shaped by environment, how mitochondria integrate stress and cell-fate signals, how mtDNA is regulated, how metabolism is rewired in disease, and whether mitochondria themselves can be harnessed therapeutically.

## Part I–Mitochondria as dynamic and environmentally responsive organelles

A recurrent theme emerging from this Research Topic is that mitochondrial function is defined not just by biochemical activity, but also by their internal organization and external context. Mitochondria form dynamic networks continuously remodeled through fission/fusion events, processes tightly linked to tissue homeostasis and disease (Sprenger and Langer, 2019; Chen et al., 2023). The review by Zhang et al. synthesizes how mitochondrial dynamics in cardiomyocytes coordinate energy production, quality control, and survival signaling. Healthy hearts adapt to changing workload and stress via fission and fusion, whereas, in disease, disturbed fission-fusion balance can produce fragmented or hyperfused mitochondrial networks that are bioenergetically inefficient, oxidatively stressed, and injury-prone. Importantly, mitochondrial dynamics are not merely descriptive features of cardiac pathology; they are mechanistically involved in disease progression and offer therapeutic targets although the context-dependent pleiotropy of fission/fusion regulators complicates translation into interventions. Perra et al. extend this theme by emphasizing that mitochondrial behavior is shaped not only by intrinsic biology but also by experimental context. Their study shows that culture configuration, oxygen availability, and model design can markedly influence mitochondrial readouts *in vitro*. More broadly, this underscores that mitochondrial phenotypes emerge from the interplay between cell-intrinsic programs and the external constraints imposed by the microenvironment and experimental systems. Together, these studies show that mitochondrial biology is shaped by the interplay between intrinsic network dynamics and the surrounding cellular environment.

## Part II–Mitochondria as stress-sensing and cell-fate decision hubs

A second major theme from this Research Topic is that mitochondria occupy a central position at the intersection of stress signaling and cell-fate control, integrating pro-survival and pro-death cues (Glover et al., 2024). Petit et al. refine this idea by focusing on a cardiolipin platform at the mitochondrial outer membrane, where caspase-8 and BID generate truncated BID (tBID) driving BAX/BAK oligomerization, mitochondrial outer membrane permeabilization, cytochrome c release, cristae remodeling, ROS production, and bioenergetic collapse. Beyond apoptosis, the article describes how BID shapes necroptosis, pyroptosis, and ferroptosis, positioning mitochondria as multi-death integrators via lipid/protein assembly. Therefore, targeting mitochondrial membrane biology may influence multiple death pathways. On the other hand, Huang and colleagues extend the stress-signaling perspective into the setting of age-related sarcopenia, bridging a key gap: treating mitochondrial dysfunction, impaired mitophagy, oxidative stress, chronic inflammation, hormonal decline, and senescence as an interconnected network rather than parallel defects. Focusing on mitochondrial quality control, this framework links AMPK/SIRT1/PGC-1α and mTORC1 signaling, together with PINK1/Parkin, BNIP3/NIX, and FUNDC1 pathways, to mitochondrial biogenesis, mitophagy, oxidative stress, and muscle maintenance. By combining these mechanisms with diagnostics and therapies, the authors move the field beyond descriptive aging phenotypes toward a more coherent model of disease biology. They also point toward an important future direction: biomarker-driven and precision-oriented strategies that can stratify sarcopenia, not only by function and muscle mass, but by the underlying mitochondrial defects sustaining disease progression.

## Part III–Mitochondrial metabolic signaling

Mitochondrial regulation extends beyond classical bioenergetics, encompassing multiple layers of metabolic signaling that range from metabolites to the mitochondrial genome itself. Metabolites such as NAD^+^ and TCA cycle intermediates function as signaling hubs that couple metabolic state to redox balance, gene regulation, and cellular responses (Chandel, 2015; Imai and Guarente, 2014), partly by shaping epigenetic landscapes through metabolite-dependent enzymes (Li et al., 2018). In parallel, mitochondrial DNA (mtDNA), long considered epigenetically inert, is now increasingly recognized as dynamically regulated, although the abundance, distribution, and functional relevance of these modifications remain debated (Shock et al., 2011; Devall et al., 2017). D’Alessandro et al. address a key question in Parkinson’s disease: whether restoring mitochondrial NAD^+^ regeneration is sufficient to rescue Complex I dysfunction in dopaminergic neurons. Using MCI-Park mice and mitochondria-targeted LbNOX, they disentangle redox balance from electron transport function. Despite partially restoring the mitochondrial NAD+/NADH balance, they observed no improvement in motor behavior, tyrosine hydroxylase levels, or dopaminergic neuron survival. These negative results are highly informative, demonstrating that preserving redox balance alone does not reconstitute complex I-dependent physiology *in vivo*. Moreover, Lu and colleagues reframe TCA metabolites (citrate, succinate, fumarate, α-ketoglutarate, and itaconate) as signaling hubs across lung diseases. Beyond metabolism, these metabolites can regulate inflammation, hypoxia responses, immune activation, vascular remodeling, and therapy resistance. This work integrates metabolic reprogramming into a translational framework for biomarkers and targeted interventions, reframing the TCA cycle as a disease-relevant signaling network. Finally, Shock and colleagues provide evidence that mammalian mitochondria harbor active machinery for both writing (mtDNMT1 and DNMT3b) and erasing (TET-like hydroxymethylase activity) cytosine methylation on mtDNA. Interestingly, reduced promoter methylation correlates with decreased transcription of mtDNA-encoded oxidative phosphorylation genes and modest reductions in mtDNA copy number, implying that, unlike in the nucleus, mtDNA methylation may support transcription. Together, these studies illustrate mitochondrial regulation as an integrated system spanning membrane dynamics, genome control and metabolite signaling, while emphasizing the importance of mechanistic precision in defining both the potential and limits of mitochondrial interventions.

## Part IV–Mitochondrial transplantation and inter-organelle transfer

Perhaps the most translationally provocative theme in this Research Topic is the idea that mitochondria themselves may become therapeutic agents. Mitochondrial transplantation builds on recognition that mitochondria can move between cells and alter recipient-cell behavior (Islam et al., 2012; Kitani et al., 2014). In principle, delivery of healthy mitochondria could bypass intrinsic defects in oxidative phosphorylation, redox balance, or quality control, offering a direct organelle-level intervention. Yet despite growing interest, the field remains scattered across experimental systems and disease contexts (Lightowlers et al., 2020). Chengze Liang and colleagues provide a timely and comprehensive review that helps bring this fragmented literature into focus, particularly in the settings of ischemia-reperfusion injury across cardiac, cerebral, hepatic, pulmonary, and renal tissues. A major strength of the article is its integration of mechanisms, methodology, organ-specific evidence, and translational challenges within a single framework, moving beyond proof-of-concept efficacy and clarifying the practical barriers to clinical implementation. Extending this perspective, work by He et al. highlights mitochondrial transfer in osteoarthritis, underscoring that intercellular mitochondrial exchange may also shape chronic degenerative disease by modulating inflammation, metabolism and tissue homeostasis. Importantly, these works emphasize that mitochondrial transplantation is not a single intervention but a chain of interdependent steps, each of which may influence outcome. While preclinical studies suggest benefits such as restored bioenergetics, reduced oxidative stress, limited calcium overload, and limited cell death (particularly when delivery coincides with reperfusion) critical questions remain unresolved regarding donor source, route and timing of administration, intracellular uptake, persistence, and long-term immunological compatibility. Together, these studies reinforce a broader conceptual shift: mitochondria are not confined to the cell but can potentially function as transferable biological units with roles in both acute injury and chronic disease.

## Future perspectives

Taken together, the contributions in this Research Topic reinforce a unifying view of mitochondria as adaptive organelles that integrate structure, metabolism, signaling, and cell fate. Beyond summarizing recent advances, the Research Topic highlights key challenges for the field: (1) advanced *in vivo* tools to study mitochondrial biology with spatial and temporal precision, (2) modeling mitochondrial pathways as interconnected systems rather than isolated modules, (3) defining therapeutic parameters (timing, targeting, persistence, and safety), and (4) elevating negative and partial-rescue studies to delineate mechanistic limits and sharpen translational strategy. In this sense, “beyond energy production” is not simply a thematic label, but an organizing principle for the next phase of mitochondrial research.
